# SIRT4 Controls Acetyl-CoA Synthesis to Promote Stemness and Invasiveness of Hepatocellular Carcinoma through Deacetylating MCCC2

**DOI:** 10.7150/ijbs.99004

**Published:** 2025-04-21

**Authors:** Tianjiao Sun, Congcong Xu, Qingru Song, Xiaodan Yang, Guo An, Guogui Sun, Zhiqian Zhang

**Affiliations:** 1 Key Laboratory of Carcinogenesis and Translational Research (Ministry of Education/Beijing), Department of Cell Biology, Peking University Cancer Hospital and Institute, Beijing 100142, P.R. China.; 2 Key Laboratory of Carcinogenesis and Translational Research (Ministry of Education/Beijing), Department of Laboratory Animal, Peking University Cancer Hospital and Institute, Beijing 100142, P.R. China.; 3 Department of Chemoradiation, Affiliated Hospital of North China University of Science and Technology, Tangshan, Hebei 063000, P.R. China.

**Keywords:** hepatocellular carcinoma, cancer stem cells, SIRT4, MCCC2, Acetyl-CoA

## Abstract

SIRT4 is well-known as a tumor suppressor by controlling several metabolic pathways, although it is highly expressed in certain cancers including hepatocellular carcinoma (HCC). Here, we reported that SIRT4 was highly expressed in the voltage-gated calcium channel α2δ1 subunit-positive HCC tumor-initiating cells (TIC), and was upregulated by α2δ1-mediated calcium signaling. Moreover, the expression of SIRT4 in HCC tissues was predictive of poor prognosis of the patients. Interestingly, SIRT4 was functionally sufficient and indispensable to promote TIC properties and invasiveness of HCC cells by directly deacetylating the leucine catabolism pathway enzyme-3-methylcrotonyl-CoA carboxylase 2 (MCCC2) at K269, leading to the formation of a stable MCCC1/MCCC2 complex with robust MCCC enzymatic activity to produce more acetyl-CoA, which resulted in increased H3K27 acetylation and stem cell-like properties at doses≤2 µM. However, 10 µM acetyl-CoA was neither able to enhance H3K27 acetylation, nor to promote stem cell-like properties, while forced expression of SIRT4 in α2δ1^+^ cells resulted in retardation of tumor growth *in vivo*. Thus, SIRT4 serves as an oncogene to promote stemness and invasiveness by controlling the production of acetyl-CoA, linking α2δ1-mediated calcium signaling to SIRT4-mediated epigenetic reprogramming of HCC TICs which hold significant potential for the development of novel therapeutic strategies targeting TICs, and the dual roles of SIRT4 in HCC might be dependent on the production levels of acetyl-CoA.

## Introduction

Acetyl coenzyme A (acetyl-CoA), is a central metabolic intermediate of multiple pathways including branched-chain amino acid (BCAA) catabolism, fatty acid oxidation (FAO), pyruvate catabolism, ketolysis, and other minor biosynthesis pathways. It is required for the tricarboxylic acid cycle, and is the essential building block for fatty acid and isoprenoid biosynthesis. It also serves as an essential signaling molecule that plays critical roles in gene expression, signaling, and other cellular processes by transfer of its acetyl group to both histone and non-histone proteins, and some metabolites in response to acetyl-CoA availability 1, 2. Furthermore, dysregulation of acetyl-CoA metabolic enzymes such as ACLY and ACSS2, both at the transcriptional and post-translational levels, has been demonstrated to contribute to aberrant levels of acetyl-CoA and tumorigenesis, and targeting of these molecules for cancer treatment has been under clinical trials 1, 3-5. However, the report that global repression of acetyl-CoA synthesis could promote oncogenicity 2 indicates that the synthesis pathway and usage of acetyl-CoA in cancer is far from being well characterized.

3-methylcrotonyl-CoA carboxylase 2 (MCCC2) interacts with MCCC1 to form a heterododecamer enzymatic complex that catalyzes the ATP-dependent carboxylation of 3-methylcrotonyl-CoA to form 3-methylglutaconyl-CoA which can be further decomposed into acetoacetate and acetyl-CoA in the leucine catabolic pathway 6, 7. High expression of MCCC2 has been found in several cancers of prostate, colorectal, and breast origins, and has been implicated in playing important roles in tumor cell proliferation and metastasis 8-10. Although post-translational modifications of 3-methylcrotonyl-CoA carboxylase, mainly the MCCC1 subunit, were found to be a key factor for the complex formation between MCCC1 and MCCC2 and for the catalytic activity in leucine metabolism and insulin secretion, which were regulated by SIRT4 7, it remains to be determined whether the dynamic regulation of post-translational modifications of MCCC in response to oncogenic signaling cues plays any roles in cancer.

SIRT4 is a mitochondrial member of sirtuins (SIRTs), a highly conserved class of nicotinamide adenine dinucleotide (NAD^+^)-dependent lysine deacylases that remove post-translational acyl modifications from various cellular substrates. It was initially identified as an NAD^+^-dependent ADP-ribosyltransferase, but recent studies have revealed its potent deacetylase activity 11, 12. Through these enzymatic and non-enzymatic activities, SIRT4 tightly regulates various metabolic events, and its dysregulation is associated with diverse diseases, including type 2 diabetes, non-alcoholic fatty liver disease, obesity, and cancer 13-15. While some studies have demonstrated that SIRT4 can suppress tumors by regulating glutamine metabolism 16-18, others have suggested its oncogenic role and observed its high expression in certain types of cancer 19, 20. Despite these observations, the precise mechanisms underlying the tumor-promoting effects of SIRT4 remain poorly understood.

The auxiliary subunit α2δ1 of voltage-gated calcium channels (VGCC) mediates the influx of Ca^2+^ into cells to activate a cascade of calcium signaling which plays essential roles in a variety of cellular processes including gene transcription, cell metabolism, proliferation, motility, cell death and survival 21, 22 and the isoform 5 of α2δ1 subunit was characterized as both a surface marker and a therapeutic target for tumor-initiating cells (TICs) of a variety of cancers including hepatocellular carcinoma (HCC) 23-25, one of the most prevalent forms of malignancies and a leading cause of cancer-related mortality worldwide 26, 27. Here, we report that SIRT4, which is up-regulated by α2δ1-mediated Ca^2+^ signaling, enhances the production of acetyl-CoA to promote the stemness and invasiveness of HCC by deacetylating MCCC2 at K269, which facilitates the complex formation with MCCC1, and thus enhances its MCCC enzymatic activity.

## Materials and methods

### Cell lines and cell culture

Human HCC cell line Huh-7 originated from the Japan Society for the Promotion of Science (Tokyo, Japan), PLC/PRF/5 cell line was obtained from the American Type Culture Collection (ATCC, Manassas, VA). The Hep-11 and Hep-12 cell lines were generated by primary culture as described in our earlier report 28. All cell lines were cultured in RPMI-1640 medium supplemented with 10% fetal bovine serum, 100 units/mL penicillin, and 100 μg/mL streptomycin (Life Technologies Corporation, Grand Island, NY) in a humidified atmosphere of 5% CO_2_ at 37 °C. The cell lines were authenticated using polymorphic short tandem repeat loci analysis and were free of mycoplasma contamination.

### Protein extraction and western blot analysis

Total protein was extracted by lysing cells or tissues in 1×Laemmli sample buffer. Proteins were separated by sodium dodecyl sulfate-polyacrylamide gel electrophoresis (SDS-PAGE) and transferred onto polyvinylidene fluoride (PVDF) membranes (Millipore, Billerica, CA). After blocking with 5% non-fat milk in TBST, membranes were incubated with the primary antibodies listed in Table S1. The HRP-conjugated secondary antibodies were purchased from Jackson ImmunoResearch Laboratories Inc. (West Grove, PA). Signals were detected with Immobilon™ Western Chemiluminescent HRP substrate (Millipore).

### Vector construction and lentivirus packaging

The open reading frames of SIRT4 and MCCC2 were polymerase chain reaction amplified from cDNAs that were reverse-transcribed from total RNAs extracted from Hep-12 cells and were subcloned into pcDNA3.0, and/or lentiviral shuttle vector plenti6 (Thermo Fisher Scientific, Waltham, MA) using standard DNA recombinant technique. For all the mutant constructs, respective point mutations were introduced using overlapped polymerase chain reaction with primers harboring the mutations and were cloned into vectors. The sgRNA-resistant wild-type and mutant MCCC2 constructs were further made by replacing the sgRNA targeting sequence with synonymous codons. For the SIRT4 and MCCC2 shRNA or sgRNA constructs, synthetic target oligos were cloned into PSIH-H1-Puro or lentiCRISPRv2 vectors. All the primers used were listed in Table S2, and the constructs were validated by sequencing. Lentiviral particles were produced in 293FT cells (Thermo Fisher Scientific) as described 25.

### Spheroid formation assay

Sphere formation assay was carried out in ultralow attachment 96-well plates (Corning Incorporated Life Sciences, Acton, MA) by plating 100 cells per well in DMEM/F-12 serum-free medium (Life Technologies Corporation) supplemented with 1 × B27 (Life Technologies Corporation), 20 ng/mL of basic fibroblast growth factor, 20 ng/mL of epidermal growth factor, 10 ng/mL of hepatocyte growth factor (Peprotech, Rocky Hill, NJ), and 1% methylcellulose (Sigma-Aldrich, St Louis, MO). After 2-3 weeks of cultivation, the number of spheres was counted under a stereomicroscope (Olympus, Tokyo, Japan).

### Co-immunoprecipitation (Co-IP) assay

Cells were harvested and washed with ice-cold PBS, followed by incubation with lysis buffer (1% NP-40, 150 mM NaCl, 5 mM EDTA, and 50 mM Tris·HCl, pH7.4) on ice for 1 h and then centrifuged to collect protein lysates. One-tenth volume of lysate was taken out as input, and the remaining lysates were incubated with primary antibodies, or control IgG at 4 °C overnight. Then, 50 μL of pre-washed protein A/G beads (Cytiva, Uppsala, Sweden) were added to the complex and incubated on a rotator for 6-8 h at 4 °C. After washing with lysis buffer for 3 times, the proteins binding to the beads were eluted with 1×Laemmli sample buffer, and were separated by SDS-PAGE.

For the Flag- or HA-tagged proteins, cell extracts were incubated with anti-Flag M2 affinity gel (Sigma-Aldrich) or anti-HA affinity gel (Sigma-Aldrich), respectively, overnight at 4 ℃, and the precipitated proteins were eluted following the respective vendors' recommendations.

### Flow cytometry

Cells were disassociated with 0.02% EDTA/PBS and re-suspended into sterile PBS and labeled with monoclonal mouse anti-α2δ1(isoform 5) antibody (1B50-1) 25 or isotype-matched mouse IgG3 conjugated with fluorescein isothiocyanate (FITC) using BD Lightning conjugation kits (Expedeon Ltd., Cambridge, UK) according to the manufacturer's protocol. After incubation at 4 ℃ for 30 min, the labeled cells were washed three times with ice-cold PBS and sorted using a FACS Aria II flow cytometer (BD Biosciences, San Jose, CA).

### Metabolomics analysis

To analyze the metabolic profile of Huh-7 cells overexpressing SIRT4, cells cultured in 10-cm dishes were extracted with 2 mL ice-cold extraction solvent (80% methanol/water) by incubation at -80 °C for 1h and centrifuged at 14,000 × g for 20 min at 4 °C. The supernatants were transferred to Eppendorf tubes and dried in a vacuum concentrator. Non-targeted metabolomic mass spectrometry analysis of the dry pellets was performed by the Metabolomics Facility at the Technology Center for Protein Sciences, Tsinghua University (Beijing, China). Metabolite pathway analysis was performed using the Pathway Analysis module of MetaboAnalyst 4.0. All metabolites were normalized to the total cell numbers used. For metabolite clustering analysis, average linkage hierarchical clustering was performed in the Statistical Analysis module of MetaboAnalyst 4.0 using Euclidian distance as a similarity metric.

### *In vitro* deacetylation assay

Flag-tagged SIRT4^WT^, SIRT4^H161Y^ mutant and MCCC2 proteins were purified from 293FT cells (Thermo Fisher Scientific) transiently transfected with respective expression constructs by immunoprecipitation using anti-Flag M2 affinity gel (Sigma-Aldrich). For the *in vitro* MCCC2 deacetylation assay, purified MCCC2 was incubated with SIRT4^WT^, or SIRT4^H161Y^ in the presence or absence of NAD^+^ in a buffer containing 50 mM Tris·HCl, pH 8.0, 4 mM MgCl_2_, 50 mM NaCl, 0.5 mM DTT at 37 °C for 1 h, followed by Western blotting using acetylated MCCC2-K269 antibody.

### Methylcrotonyl-CoA carboxylase activity assay

Mitochondria were isolated from cells using ExKine™ Mitochondrion Extraction Kit (Abbkine Scientific Co., Ltd, Wuhan, China) according to the manufacturer's instruction. The never-frozen mitochondrial pellets were resuspended in 100 μL permeabilization buffer (105 mM K-MES, pH 7.1, 30 mM KCl, 10 mM KH_2_PO_4_, 5 mM MgCl_2_, 0.5 mg/mL BSA, 50 mg/mL digitonin) and incubated on ice for 5 min. To 10 μL of permeabilized mitochondria, we added 100 μL MCCC reaction mixture containing 105 mM K-MES, pH 7.1, 30 mM KCl, 10 mM KH_2_PO_4_, 5 mM MgCl_2_, 0.5 mg/mL BSA, 2 mM ATP, 0.1 mM DTT, 0.3 mM methylcrotonyl-CoA (Sigma-Aldrich) and 3μCi NaH[^14^C]O_3_ (PerkinElmer, Inc., Waltham, MA). The reaction was carried out at 37 °C for 30 min and stopped with HCl. The unreacted NaH[^14^C]O_3_ was evaporated by placing Eppendorf tubes in the radiation fume hood at 60 °C overnight. The dry sediment was resuspended in 0.5 mL DI-H_2_O and analyzed on a scintillation counter. Counts per minute (cpm) were normalized to protein content.

### Acetyl-coenzyme A measurement

The content of acetyl-CoA was determined with a PicoProbe AcCoA assay kit (Abcam, Cambridge, MA), according to the manufacturer's instruction. In brief, cultured cells were deproteinized by perchloric acid precipitation, followed by adding acetyl-CoA Quencher and Quencher remover to each sample to correct the background created by free Coenzyme and succinyl-CoA. The sample was then diluted with the reaction mixes, and the fluorescence was measured using the SpectraMax® iD3 Multi-Mode Microplate Reader (Molecular Devices, LLC., San Jose, CA) with the following settings: λex 535 nm; λem 587 nm. The acetyl-CoA standard curve was made in the range of 0-100 pM and the correlation coefficient was 0.990 or higher.

### RNA-seq and data analysis

Cells were collected in TRIzol^®^ Reagent (Thermo Fisher Scientific) and sent to Mingma Technologies (Shanghai, China) to prepare RNA sequencing libraries and sequenced by Illumina Nova 6,000 sequencer (pair-end 150 bp). Raw sequence generated from Illumina Nova 6,000 was converted into fastq format files and removed the adapters with Trim galore software (v.0.6.7). Then clean reads were first aligned to the human 45S pre-rRNA and unmapped reads were collected to align to the human genome assembly version (hg19) using the HISAT2 software (v.2.1.0). Mapped reads were used to quantify the number of genes by featureCounts program (v.2.0.1). The differentially expressed genes (DEGs) were characterized using the DESeq2 package (v.1.24.0) with following parameters: |Fold Change|≥1.5 and p-value < 0.05.

### ChIP-seq and data analysis

Cells were cross-linked with 1% formaldehyde for 15 minutes at room temperature, and chromatin was fragmented to an average size of approximately 300-500 bp by sonication. Fragmented chromatin was further incubated with 5 μg of either anti-H3K27ac antibody or nonspecific rabbit IgG at 4°C overnight, and immunoprecipitated DNA was eluted and purified using a PCR purification kit (Qiagen, Valencia, CA). The immunoprecipitated DNA was ligated with adaptors, and PCR amplified with index primers to prepare ChIP-seq libraries. The libraries were sent to Novogene (Beijing, China) for sequencing on an Illumina Nova 6000 sequencer.

Raw sequencing data generated from Illumina NovaSeq was converted into fastq format files, and the adapters were removed using Trim galore software (v.0.6.7). Then the clean reads were aligned to the human genome assembly version (hg19) using the bwa-mem with M parameter (v.0.7.17). Metaplot and heatmap were generated using the deeptools software (3.3.0). BigWigAverageOverBed was used to calculate the H3K27ac occupancy around ± 2Kb of transcription start site (TSS).

### Tumorigenicity assay

For the tumorigenicity assay, cells were suspended in 100 μL of a 1:1 mix of serum-free medium and Matrigel (BD Biosciences, Bedford, MA) and subcutaneously injected into the flanks of 4- to 6-week-old female nonobese diabetic/severe combined immunodeficient (NOD/SCID) mice (Vitalriver, Beijing, China). Tumor formation was monitored weekly. For the tumor growth curves, tumors were measured every other day with calipers, and individual tumor volumes (V) were determined using the formula: V=length×width^2^ ×0.5. All animal experiments were performed in accordance with the National Institutes of Health Guide for the Care and Use of Laboratory Animals with protocols approved by the Animal Care and Use Committee of Peking University Cancer Hospital.

### Statistics

Statistical analysis was performed using GraphPad Prism 9. Data with error bars are presented as mean ± SD. Statistical testing was performed using the Two-tailed Student's *t* test unless otherwise specified. Tumorigenic cell frequency was calculated based on extreme limiting dilution analysis using the ELDA web tool at http://bioinf.wehi.edu.au/software/elda/. A p value ≤ 0.05 was considered statistically significant.

## Results

### SIRT4 is upregulated in HCC tissues and is predictive of poor prognosis of HCC patients

**Figure 1:**
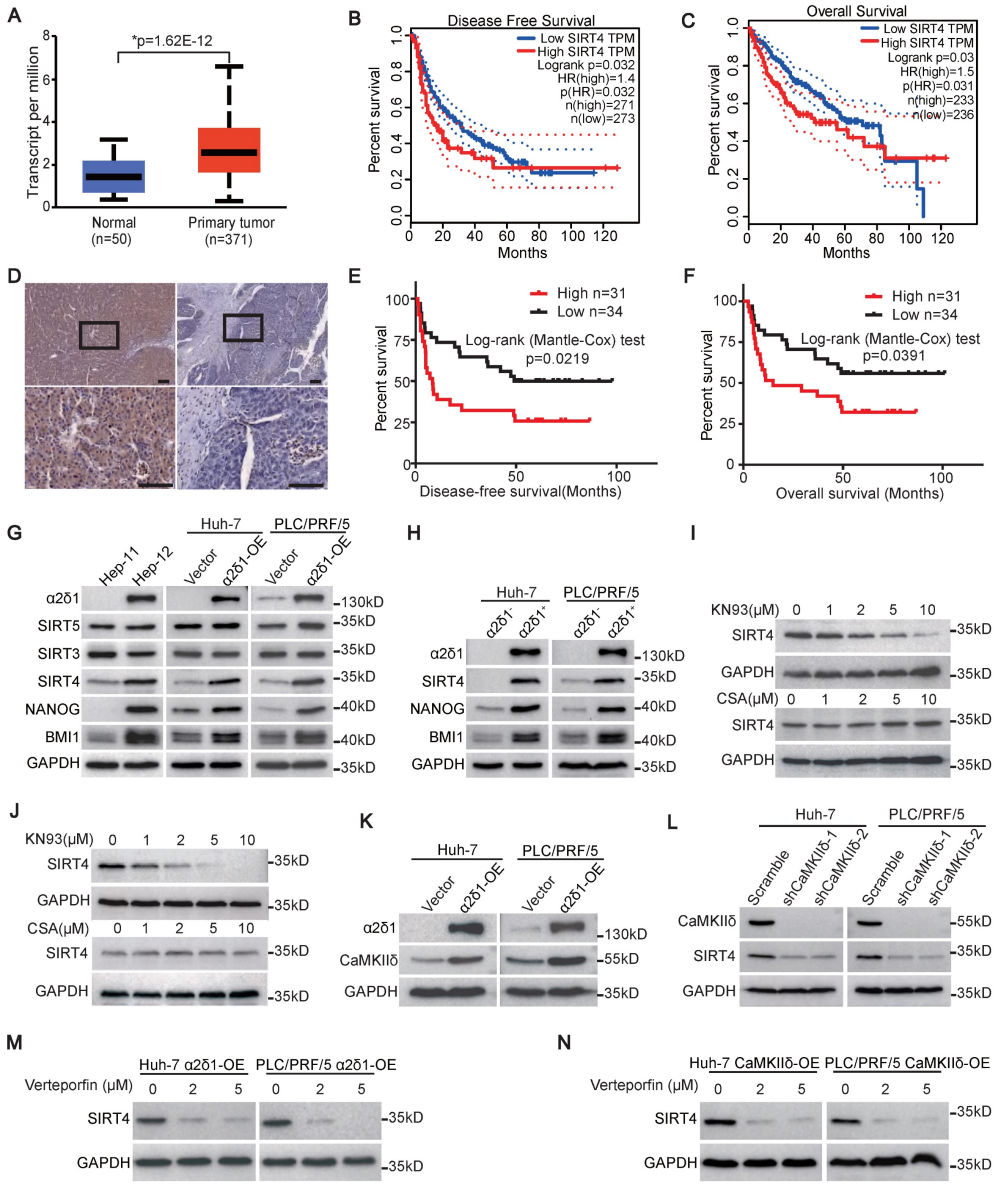
** SIRT4 is upregulated through α2δ1-mediated calcium signaling pathway and is predictive of poor prognosis of HCC patients. (A)** The expression of *SIRT4* in HCC and normal tissues from the TCGA database was analyzed using an online tool (http://ualcan.path.uab.edu/index.html). **(B and C)** Kaplan-Meier survival analysis for the expression of SIRT4 in HCC patients was performed using an online tool (http://gepia2.cancer-pku.cn/). **(D)** Representative immunohistochemical staining images showing high expression (left) and low expression (right) of SIRT4 in human HCC samples. Scale bars: 100 μm. **(E and F)** Kaplan-Meier curves showing the disease-free survival (DFS) **(E)** and overall survival (OS) **(F)** for HCC patients divided by SIRT4 staining status in cancer tissues. **(G)** Western blotting analysis of the expression of the indicated molecules in Hep-11 and Hep-12 cells, as well as Huh-7 and PLC/PRF/5 cells overexpressing (OE) α2δ1. **(H)** Western blotting results showing the expression of the indicated molecules in the α2δ1^+^ and α2δ1^-^ fractions sorted from the indicated cells. **(I and J)** Western blotting results showing the expression of SIRT4 in Huh-7 **(I)** and PLC/PRF/5 **(J)** cells after CsA or KN93 treatment for 48h at the indicated concentrations. **(K)** Western blotting results showing the expression of CaMKIIδ in the indicated cells overexpressing α2δ1. (**L**) Western blotting analysis of the expression of SIRT4 in Huh-7 and PLC/PRF/5 cells overexpressing α2δ1 after knockdown of *CaMKIIδ* by specific shRNAs. **(M and N)** Western blotting results showing the expression of SIRT4 in the indicated cells overexpressing *α2δ1* **(M)** or *CaMKIIδ* **(N)** treated with Verteporfin for 24h at the indicated concentrations.

We first analyzed the expression of *SIRT4* in HCC using datasets from the Cancer Genome Atlas (TCGA), and revealed that *SIRT4* mRNA was significantly upregulated in HCC tissues compared with adjacent paracancerous tissues (Figure 1A). Furthermore, the disease-free survival (DFS) and overall survival (OS) of those patients with high expression of *SIRT4* mRNA in HCC tissues were much shorter than those with low *SIRT4* expression (Figure 1B and C). We then performed immunohistochemistry staining using an antibody against SIRT4 and analyzed the association between SIRT4 levels and clinicopathological features in 65 cases of HCC specimens (Figure 1D). Although the expression of SIRT4 was not found to be associated with gender, age, hepatic cirrhosis, and venous invasion, a high level of SIRT4 was positively associated with large tumor size (>5 cm), high AFP level (>400μg/L), and early recurrence of HCC (Table S3). Kaplan-Meier curves indicated that high levels of SIRT4 staining were positively correlated with shorter DFS and OS of the patients (Figure 1E and F).

### SIRT4 is the only mitochondrial Sirtuin upregulated by α2δ1-mediated calcium signaling

The association of elevated SIRT4 expression with early recurrence of HCC promoted us to address whether the expression of SIRT4 was related to the TIC properties of HCC, which was proposed to be responsible for cancer recurrence 29, 30. We first detected the expression of SIRT4, along with other mitochondrial Sirtuins including SIRT3 and SIRT5, in the paired Hep-11 and Hep-12 HCC cell lines, which originated from the same patient's primary and recurrent HCC tissues and represented non-tumorigenic and TIC-enriched cell populations, respectively 28. As shown in Figure 1G, the expression of SIRT4, but not SIRT3 or SIRT5, and the stem cell-related genes BMI1 and NANOG, was much higher in the α2δ1-positive Hep-12 cell line than in the α2δ1-negative Hep-11 one. Furthermore, SIRT4 was the only mitochondrial Sirtuin that was consistently upregulated in both the Huh-7 and PLC/PRF/5 cell lines that ectopically expressed α2δ1 (Figure 1G). In addition, the expression of SIRT4 was also much higher in the α2δ1^+^ fractions than in the respective α2δ1^-^ ones purified from the Huh-7 and PLC/PRF/5 cell lines (Figure 1H).

Our previous studies have linked CaMKIIδ and Calcineurin/NFATc2 to the roles of α2δ1-mediated Ca^2+^ influx in the acquisition and maintenance of stem cell-like properties of pancreatic and non-small cell lung TICs, respectively 23, 24. Therefore, we addressed which calcium signaling pathway was involved in the upregulation of SIRT4 mediated by α2δ1 through treatment with their inhibitors. Treatment of Huh-7 and PLC/PRF/5 cells overexpressing α2δ1 with CaMKII inhibitor KN93 led to significant downregulation of SIRT4 in a dose dependent manner, whereas treatment with the calcineurin inhibitor cyclosporine (CsA) had little effect on the expression of SIRT4 (Figure 1I and J). Furthermore, *CaMKIIδ* was found to be upregulated in the Huh-7 and PLC/PRF/5 cell lines after forced expression of α2δ1 (Figure 1K), and forced expression of *CaMKIIδ* in Huh-7 and PLC/PRF/5 cells resulted in the increased expression of SIRT4, but not mitochondrial SIRT3 and SIRT5 (Figure S1A), as well as the expression of stemness related genes and enhanced sphere formation ability (Figure S1A-C). On the contrary, knockdown of *CaMKIIδ* with its specific shRNAs in α2δ1-overexpressing cells led to the downregulation of SIRT4 (Figure 1L). In support of its role in promoting TIC properties, the expression of *CaMKIIδ* at mRNA level was also positively correlated with early recurrence of HCC patients as analyzed using the dataset from GEPIA2 (Figure S1D). These data confirm that CaMKIIδ was responsible for α2δ1-mediated upregulation of SIRT4.

CaMKIIδ was demonstrated to be able to phosphorylate PKM2 at T454, leading to subsequent phosphorylation of PKM2 at Y105, which induced Yes-associated protein (YAP) nuclear localization to activate the downstream YAP signaling pathway and acquire stem-like properties 23, 31. Therefore, we treated Huh-7 and PLC/PRF/5 cells overexpressing *α2δ1* or *CaMKIIδ* with YAP inhibitor verteporfin to address if the CaMKIIδ-PKM2-YAP pathway was involved in the upregulation of SIRT4 mediated by *α2δ1.* As shown in Figure 1M and N, neither *α2δ1* nor* CaMKIIδ* could upregulate the expression of SIRT4 when the cells were treated with verteporfin.

All these data demonstrate that SIRT4 is the only mitochondrial Sirtuin that is upregulated by α2δ1-mediated calcium signaling involving the CaMKIIδ-YAP pathway.

### SIRT4 is essential for the acquisition and maintenance of HCC TIC properties

**Figure 2:**
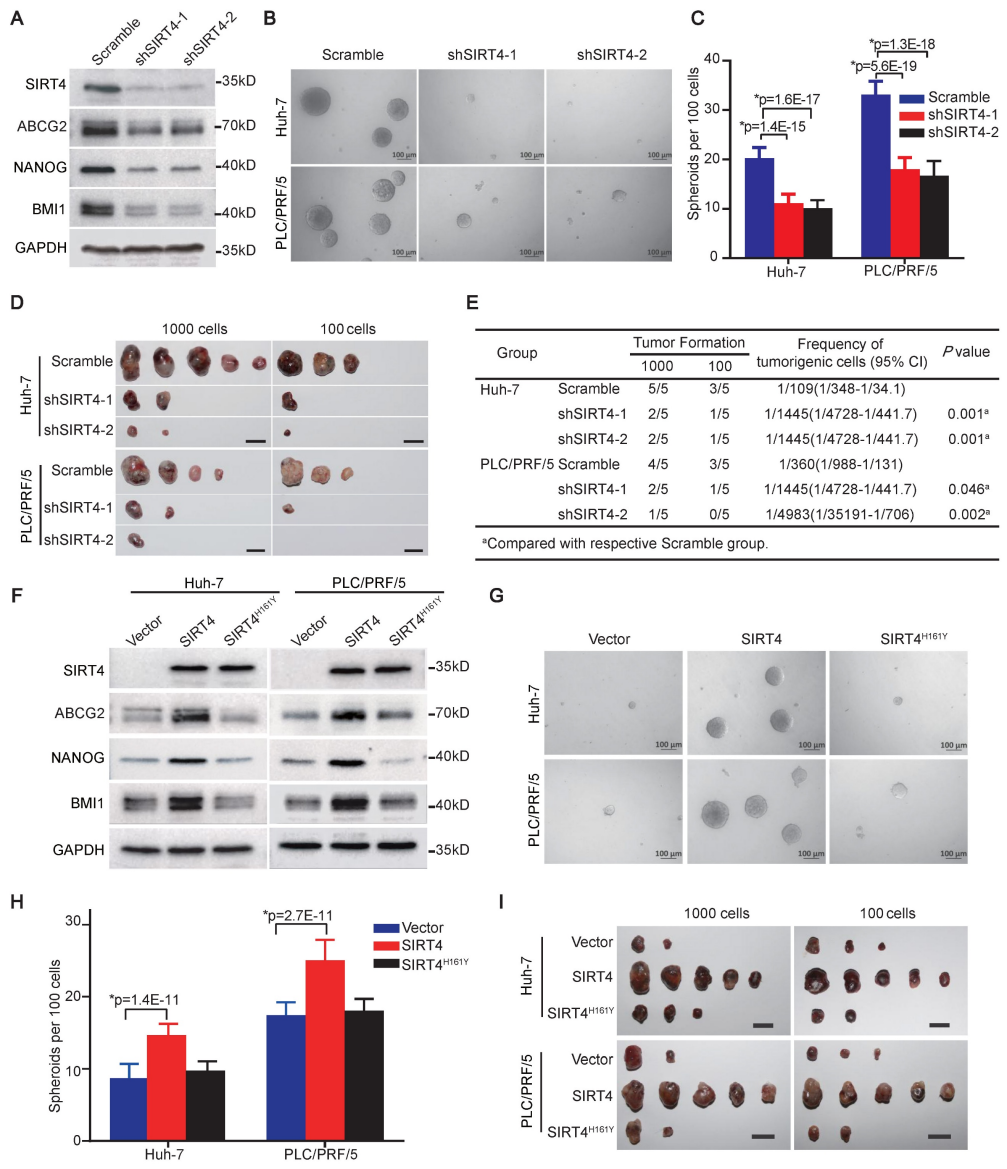
** SIRT4 is essential for the acquisition and subsequent maintenance of HCC TIC properties. (A)** Western blotting analysis of the indicated molecules in Hep-12 cells after knockdown of SIRT4 by specific shRNAs. **(B and C)** Representative phase contrast micrographs **(B)** and histograms **(C)** showing the spheroid formation ability of α2δ1^+^ subsets from the indicated sources after SIRT4 knockdown with shRNAs. 100 cells per well were plated (n = 6). Spheroids (≥100 μm) were counted under a stereomicroscope. **(D)** Tumorigenicity of α2δ1^+^ fractions purified from the indicated sources after the knockdown of SIRT4 with specific shRNAs (n=5). Bars = 1 cm. **(E)** Tumor-initiating cell frequencies of purified α2δ1^+^ fractions from the indicated sources after SIRT4 shRNA knockdown. **(F)** Western blotting results showing the expression of the indicated molecules in the α2δ1^―^ cells purified from Huh-7 and PLC/PRF/5 cell lines after SIRT4 or SIRT4^H161Y^ overexpression. **(G and H)** Phase contrast micrographs **(G)** and histograms **(H)** demonstrate spheres formed by the indicated α2δ1^―^ cells after forced expression of SIRT4 or SIRT4^H161Y^. **(I)** Tumorigenicity of the indicated α2δ1^―^ cells after forced expression of SIRT4 or SIRT4^H161Y^ (n = 5). Bars = 1 cm. Data in C and H are expressed as mean ± SD of 3 independent experiments (n = 6). *Two-tailed Student's *t* test.

We subsequently determined the functional significance of SIRT4 in α2δ1^+^ HCC TICs by knockdown its expression in Hep-12 cell line and α2δ1^+^ TICs sorted from Huh-7 and PLC/PRF/5 cell lines using shRNAs targeting SIRT4. Western blot results revealed that the expression of SIRT4, and a panel of stem cell-related molecules including ABCG2, BMI1, and NANOG was significantly suppressed in Hep-12 cells following infection with lentiviruses harboring SIRT4 shRNAs (Figure 2A). Furthermore, the spheroid formation abilities and tumorigenicity of α2δ1^+^ fractions purified from Huh-7 and PLC/PRF/5 cell lines were significantly retarded after SIRT4 knockdown (Figure 2B-E).

We then ectopically expressed SIRT4 in α2δ1^-^ cells sorted from Huh-7 and PLC/PRF/5 cell lines to address whether SIRT4 is sufficient to drive the formation of HCC TICs. Compared with the respective control cells infected with empty lentivirus, overexpression of SIRT4 in α2δ1^-^ cells resulted in elevated expression of stem cell-related genes such as ABCG2, BMI1, and NANOG (Figure 2F), along with significantly enhanced abilities to initiate spheroid formation in serum-free medium (Figure 2G and H), and increased tumor-formation capacities in NOD/SCID mice (Figure 2I, Table 1).

The 161st histidine (H) of SIRT4 has been identified as a critical site for its catalytic enzyme activity, and mutation of this site to tyrosine (Y) abolishes its enzyme activity 7. We therefore constructed a catalytically inactive SIRT4 mutant (SIRT4^H161Y^) and ectopically expressed it in α2δ1^-^ HuH-7 and PLC/PRF/5 cells to address whether the promoting effects of SIRT4 on TIC properties are dependent on its enzymatic activity. The overexpression of SIRT4^H161Y^ was neither able to upregulate the expression of stem cell-related genes (Figure 2F), nor affect the spheroid-forming ability or tumorigenicity of these cells (Figure 2G-I, Table 1).

These data demonstrate that SIRT4 is sufficient to reprogram α2δ1^-^ cells into TICs, which depends on its enzymatic activity, and is essential for the subsequent maintenance of stem cell-like properties of α2δ1^+^ HCC TICs.

### SIRT4 controls the synthesis of acetyl-CoA

**Figure 3:**
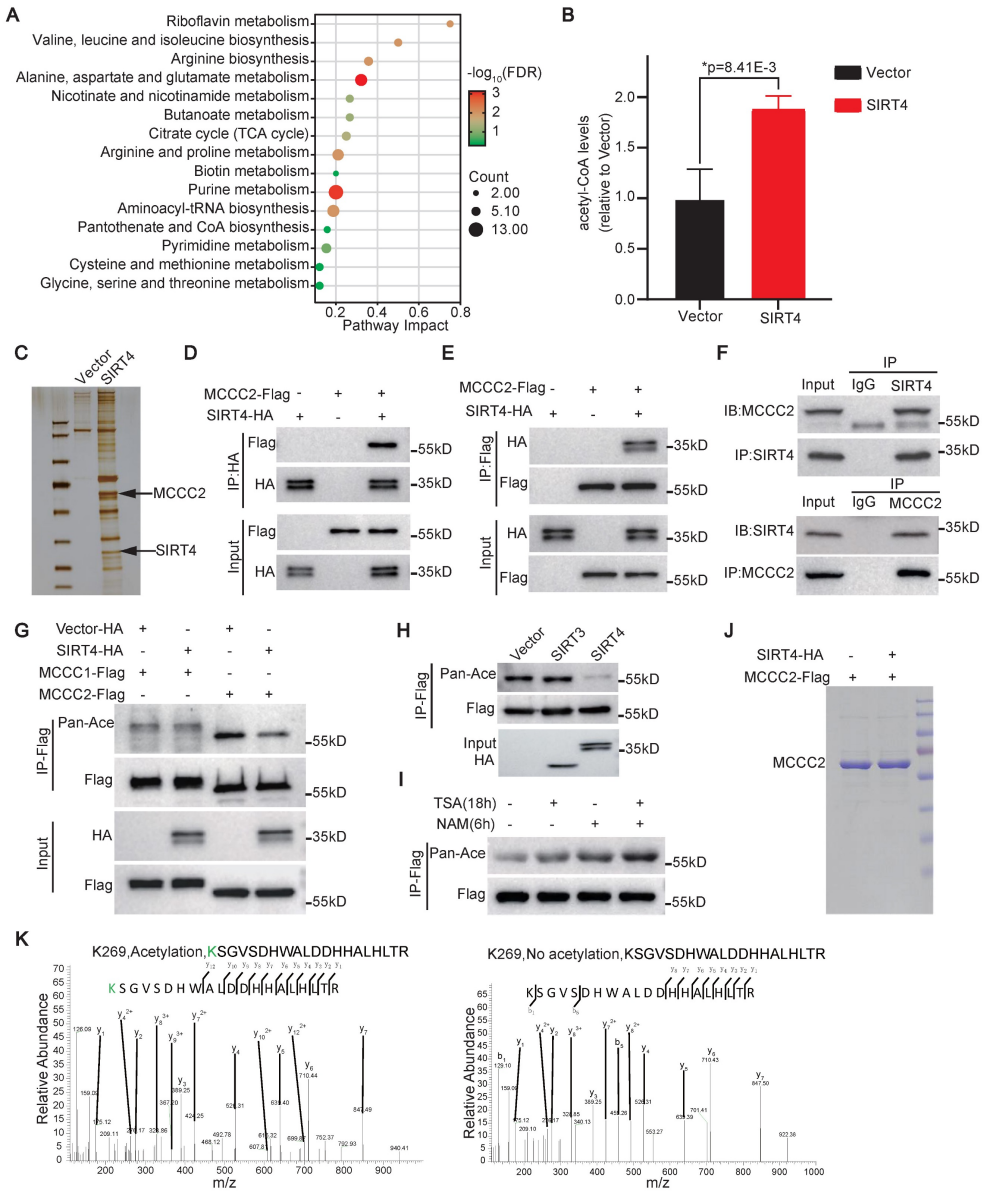
** SIRT4 enhances acetyl-CoA synthesis and deacetylates MCCC2. (A)** Pathway analysis of differentially enriched metabolites in Huh-7 cells overexpressing SIRT4 compared with the control cells infected with vector alone lentivirus. **(B)** The levels of acetyl-CoA, as quantified by LC-MS, in Huh-7 cells after forced expression of SIRT4 (n=3), *Two-tailed Student's *t* test.** (C)** SDS-PAGE analysis of immunoprecipitated products with anti-Flag M2 affinity gel in 293FT cells transiently transfected with SIRT4-Flag construct. **(D and E)** Western blotting analysis of the immunoprecipitated products of anti-HA **(D)** and anti-Flag **(E)** in 293FT cells transiently transfected with the indicated constructs using the indicated antibodies. **(F)** Western blotting analysis of the immunoprecipitated products with the indicated antibodies in Hep-12 cells. **(G)** Western blotting analysis of the acetylation levels of MCCC1 and MCCC2 in the cell lysates of 293FT cells transiently transfected with the indicated constructs using pan anti-acetylated lysine antibody. **(H)** Western blotting results showing the acetylation levels of MCCC2-Flag in 293FT cells co-transfected MCCC2-Flag with SIRT4-HA or SIRT3-HA. **(I)** The acetylation levels of MCCC2-Flag in 293FT cells overexpressing MCCC2-Flag after treatment with deacetylase inhibitors TSA (10 μM) or NAM (5 mM) as demonstrated by Western blotting using pan anti-acetylated lysine antibody. **(J)** MCCC2-Flag was immunoprecipitated from the cell lysates of 293FT cells co-transfected with MCCC2-Flag and SIRT4, or vector alone constructs, and was separated by SDS-PAGE for acetylation analysis by mass spectrum.** (K)** Mass spectrometry analysis of the acetylation sites of MCCC2-Flag immunoprecipitated in **(I)**.

To search for the molecular mechanism(s) underlying the role of SIRT4 in promoting the stem cell-like properties of HCC TICs, we first performed metabolomic analysis of Huh-7 cells overexpressing SIRT4. Compared with the vector alone control cells, a total of 204 metabolites were differentially detected in the SIRT4 overexpressing cells, including 120 metabolites upregulated and 84 metabolites downregulated (Table S4). KEGG pathway mapping for all differential metabolites analysis showed that SIRT4 overexpression resulted in the dysregulation of multiple intracellular metabolic pathways, including those related to amino acid metabolism, nicotinate and nicotinamide metabolism, butanoate metabolism, and citrate cycle (TCA cycle) (Figure 3A). In particular, the level of acetyl-CoA, the intermediate metabolite of BCAA and TCA cycle, was found to be significantly increased after SIRT4 overexpression (Figure 3B). These data suggest that SIRT4 may control the synthesis of acetyl-CoA.

### SIRT4 directly deacetylates MCCC2 at K269

We then carried out immunoprecipitation assay with anti-Flag M2 beads in the cell lysates of 293FT cells transiently transfected with SIRT4-Flag constructs to identify potential substrate(s) of SIRT4 that are involved in its roles in promoting the stemness and metabolism of HCC. Compared with the cells transfected with an empty control vector, the SIRT4-Flag cell lysates precipitated SIRT4 itself as expected, as well as several other candidates, including MCCC2, which plays a critical role in BCAA metabolism and HCC development 32, as revealed by mass spectrometry analyses of the bands indicated in Figure 3C. The interaction between SIRT4 and MCCC2 was further verified through co-immunoprecipitation (Co-IP) followed by Western blot assays in 293FT cells transiently transfected with SIRT4-HA and MCCC2-Flag. As shown in Figure 3D and E, MCCC2-Flag was co-precipitated by the anti-HA antibody, and SIRT4-HA was also co-precipitated by the anti-Flag antibody. The physiological binding between SIRT4 and MCCC2 was finally validated by performing Co-IP in Hep-12 cells using antibodies against SIRT4 and MCCC2. As expected, MCCC2 was immunoprecipitated by the antibody against SIRT4, and vice versa (Figure 3F), indicating that these two proteins bind to each other endogenously. Moreover, the overall lysine acetylation level of MCCC2-Flag precipitated from 293FT cells co-transfected with MCCC2-Flag and SIRT4 was much lower than that co-transfected MCCC2-Flag with control plasmid, whereas ectopic expression of SIRT4 had little effect on the overall lysine acetylation level of transfected MCCC1-Flag (Figure 3G). In addition, co-transfection of MCCC2 with SIRT3, which exhibits robust deacetylase activity in mitochondria, led to little change in the overall lysine acetylation level of MCCC2 (Figure 3H), further supporting that the deacetylation of MCCC2 is SIRT4 specific. Consistent with these findings, treatment of 293FT cells overexpressing MCCC2-Flag with nicotinamide (NAM), an inhibitor of the SIRT family deacetylases, resulted in a much more pronounced increase of acetylation level of MCCC2-Flag than the treatment with trichostatin A (TSA), an inhibitor of histone deacetylases (HDACs) I and II (Figure 3I), suggesting that MCCC2 is primarily deacetylated by a member of the SIRT deacetylase family.

**Figure 4:**
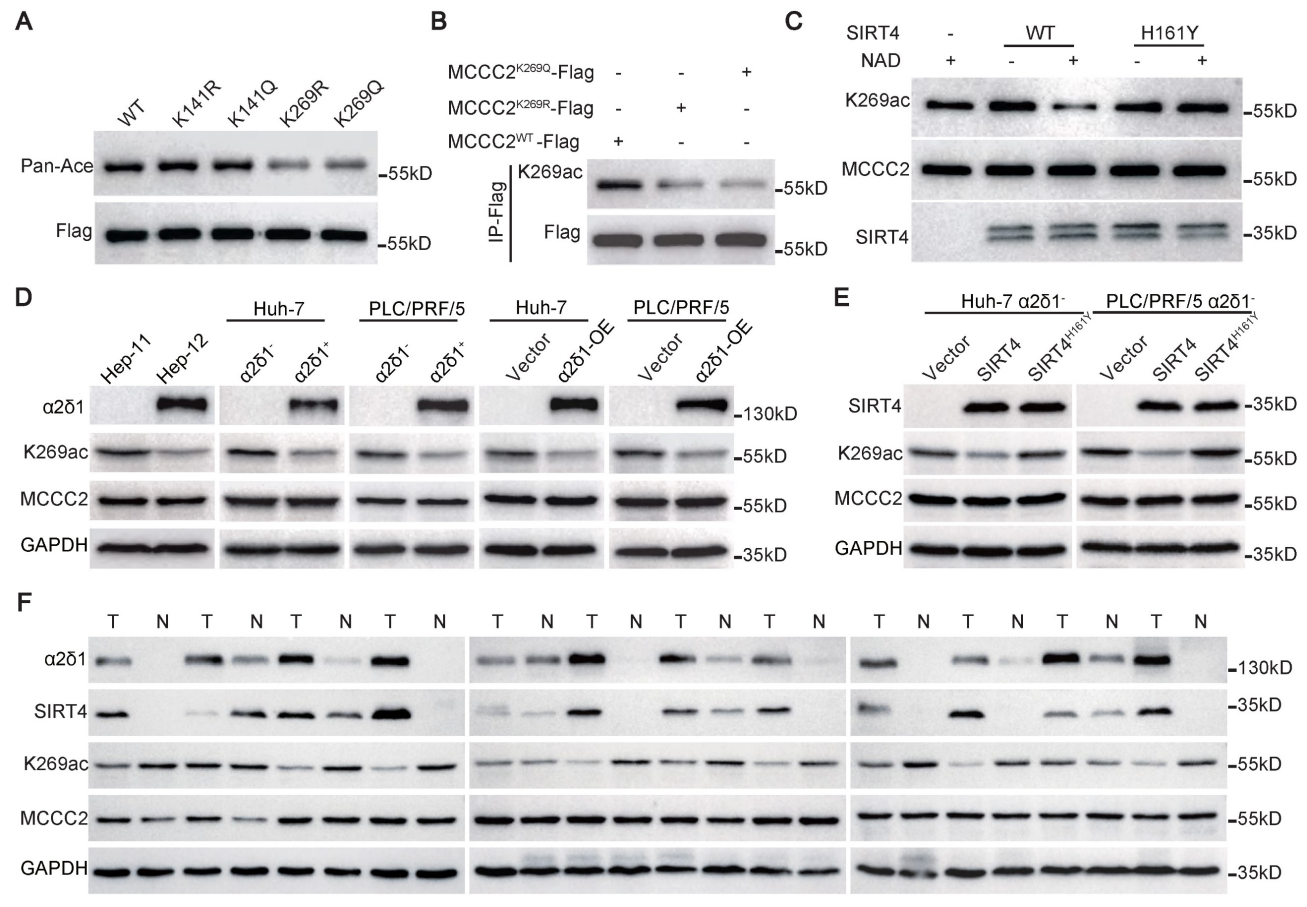
** SIRT4 directly deacetylates MCCC2 at K269. (A)** Western blotting results showing the acetylation levels of the indicated MCCC2 mutants in 293FT cells transiently transfected with the indicated constructs using pan anti-acetylated lysine antibody. **(B)** Western blotting analysis of the acetylation level of MCCC2 in the cell lysates of 293FT cells transiently transfected with the indicated constructs using the acetylation site-specific antibody(K269ac). **(C)** Western blotting analysis of in vitro deacetylation assay products with the indicated antibodies. Purified MCCC2-Flag was incubated with purified SIRT4^WT^-Flag or SIRT4^H161Y^-Flag in the presence or absence of NAD. **(D and E)** The levels of total MCCC2 and the acetylated MCCC2-K269 were determined by western blotting in the indicated cells. **(F)** Western blotting results showing the expression of the indicated molecules in 12 pairs of HCC (T) and adjacent normal (N) tissues.

Mass spectrometry analysis for the acetylation sites of MCCC2 identified that two acetylation sites, K141 and K269, presented only in MCCC2 immunoprecipitated from the cells transfected with control plasmid, but not in that from SIRT4 transfected cells (Figure 3J and K, Figure S2A). Subsequent mutation of each of the two lysine residues to arginine (R, deacetylated mimetic), or glutamine (Q, acetylated mimetic) verified that only mutation of K269 reduced the acetylation levels of MCCC2 (Figure 4A), indicating that K269 is a major acetylation site of MCCC2 under tested conditions. We then generated a site-specific antibody against acetylated MCCC2 at K269 (Ace-K269) (Figure S2B). Western blot analysis showed that this antibody could detect exogenous MCCC2 immunoprecipitated from the cells transiently transfected with MCCC2-Flag, but it only reacted with the K269R and K269Q mutants at levels just above the background (Figure 4B). Further *in vitro* deacetylase assay using purified MCCC2 and SIRT4 demonstrated that SIRT4 could deacetylate MCCC2-K269 directly, whereas the deacetylase-dead mutant SIRT4^H161Y^ resulted in little change in the acetylation level of MCCC2-K269 (Figure 4C).

Further Western blot using the antibody against Ace-K269 confirmed that the acetylation levels of MCCC2-K269 were much lower in Hep-12 cell line, the sorted α2δ1^+^ and α2δ1-OE Huh-7 and PLC/PRF/5 cells than in Hep-11 cell line, α2δ1^-^ subpopulations and their respective vector alone control cells, respectively, whereas the total MCCC2 remained the same (Figure 4D). Ectopic expression of SIRT4 in α2δ1^-^ Huh-7 and PLC/PRF/5 cells also led to a significantly decreased acetylation level of MCCC2-K269, whereas the deacetylase-dead mutant SIRT4^H161Y^ had little effect on the acetylation level of MCCC2-K269 (Figure 4E). Additionally, the level of acetylated MCCC2-K269, which was downregulated in HCC tissues compared with the matched paracancerous tissues, was negatively correlated with those of SIRT4 and α2δ1 as demonstrated in 12 paired HCC tissues and adjacent normal tissues by Western blot (Figure 4F).

Collectively, these data demonstrate that MCCC2 is a bona fide substrate for SIRT4 which deacetylates it directly at K269 in α2δ1^+^ HCC TICs.

### Deacetylated MCCC2 at K269 promotes the stem cell-like properties of HCC TICs

**Figure 5:**
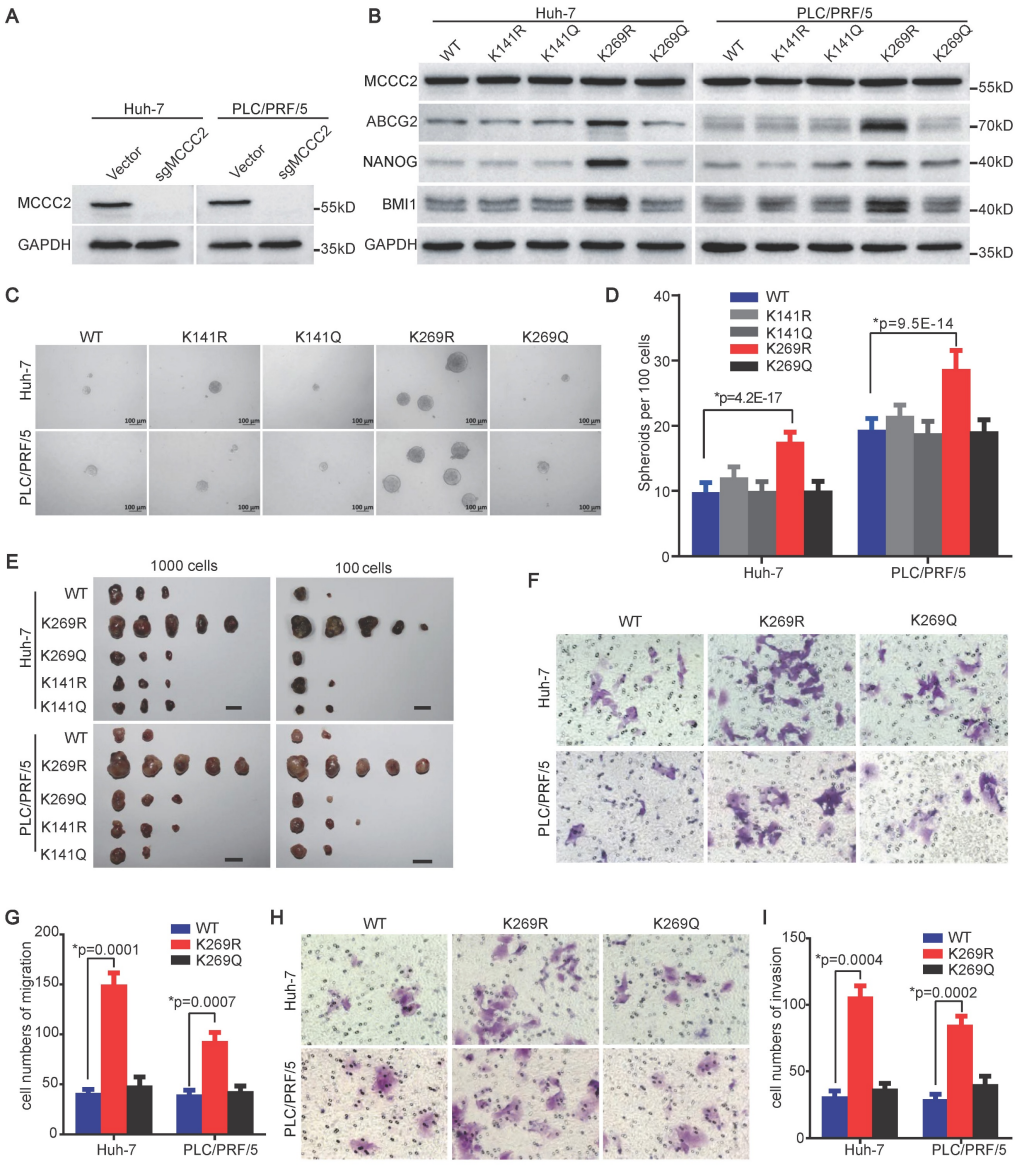
** Deacetylated MCCC2 at K269 promotes the stem cell-like properties of HCC TICs. (A)** Endogenous MCCC2 knockout efficacy in the indicated cells was verified by western blotting. **(B)** Western blotting results showing the expression of the indicated molecules in MCCC2-KO Huh-7 and PLC/PRF/5 cells overexpressing the indicated constructs that are sgRNA-resistant. **(C and D)** Representative phase contrast images **(C)** and Histograms** (D)** showing the sphere-forming ability of the indicated MCCC2-KO cells overexpressing the indicated sgRNA-resistant constructs. Scale bars: 100 μm.** (E)** Tumorigenicity of the indicated MCCC2-KO cells overexpressing the indicated sgRNA-resistant constructs.** (F-I)** Cell migration (**F and G**) and invasion (**H and I**) assays of the indicated MCCC2-KO cells overexpressing the indicated sgRNA-resistant constructs. Data represent mean ± SD of 3 independent experiments. *Two-tailed Student's* t* test.

To test if the deacetylation of MCCC2 at K269 contributes to the acquisition of stem cell-like properties of HCC TICs, we reconstituted the expression of sgRNA-resistant wild type (WT), deacetylated mimetic mutants (K141R and K269R), and acetylated mimetic mutants (K141Q and K269Q) in Huh-7 and PLC/PRF/5 cell lines with endogenous MCCC2 knocked out using CRISPR/Cas9 technique (Figure 5A and B). Compared with the cells expressing WT MCCC2, forced expression of K269R in the two cell lines resulted in upregulation of stem cell-related genes detected, including ABCG2, BMI1, and NANOG (Figure 5B), enhanced spheroid formation abilities (Figure 5C and D), and increased tumorigenicity (Figure 5E, Table 2), whereas the expression of K141R, K141Q and K269Q led to negligible change on the stemness of these cells (Figure 5B-E, Table 2). Importantly, ectopic expression of the K269R mutant in Huh-7 and PLC/PRF/5 cells led to a significantly increased number of cells migrating through membrane, as well as invading through Matrigel, compared with the respective cells expressing WT, and K269Q constructs (Figure 5F-I). These data demonstrate that the deacetylation of MCCC2 at K269 promotes the acquisition of stem cell-like properties, migration and invasiveness capacities of HCC cells.

### The deacetylation of MCCC2 at K269 increases its enzymatic activity and acetyl-CoA production by facilitating its interaction with MCCC1

**Figure 6:**
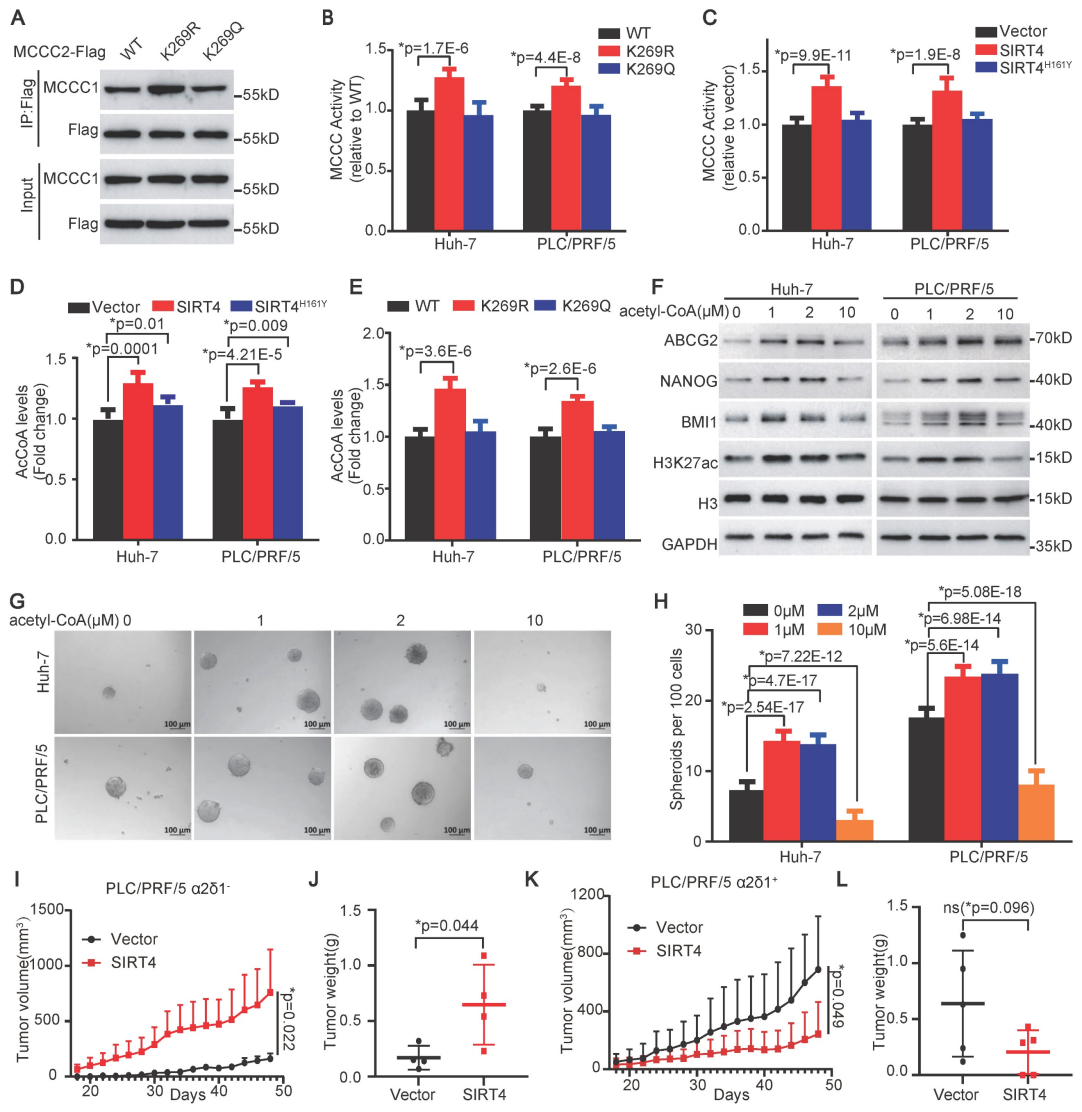
** SIRT4 enhances MCCC enzymatic activity and acetyl-CoA production by deacetylating MCCC2 at K269. (A)** Western blotting results showing the interaction of the indicated MCCC2 mutants with MCCC1. **(B and C)** MCCC activity was measured in the indicated cells. **(D and E)** Measurement of the acetyl-CoA levels in the indicated cells.** (F)** Western blotting results showing the expression of the indicated molecules in the SLO (0.8 μg/mL)-pretreated cells after treatment with acetyl-CoA for 48h at the indicated concentrations. **(G and H)** Representative phase contrast images** (G)** and Histograms **(H)** showing the sphere-forming ability of the SLO (0.8 μg/mL)-pretreated cells after acetyl-CoA treatment at the indicated concentrations. **(I and J)** Growth curves **(I)** and the weights **(J)** of the tumors formed by α2δ1^-^ PLC/PRF/5 cells overexpressing SIRT4 or empty vector in NOD/SCID mice (n=4). **(K and L)** Growth curves **(K)** and the weights **(L)** of the tumors formed by α2δ1^+^ PLC/PRF/5 cells overexpressing SIRT4 or empty vector. (n=5). Bars = 1 cm. Data represent mean ± SD of 3 independent experiments. *Two-tailed Student's *t* test.

Previous studies well established that SIRT4 interacted with BCAA catabolic enzyme MCCC1, of which the deacylation led to enhanced formation of heterododecamer catalytic enzyme complex with elevated MCCC1 enzymatic activity 7, 33. Our finding that SIRT4 increased the level of acetyl-CoA and deacetylated MCCC2 promoted us to address whether the deacetylation of MCCC2 at K269 had similar effects. Western blot analysis of the MCCC2 immunoprecipitation products from the endogenous MCCC2-KO cells ectopically expressing MCCC2^WT^, MCCC2^K269R^, and MCCC2^K269Q^ with the antibody against MCCC1 revealed that the K269R mutant could pull down much more MCCC1 than the WT, K269Q did (Figure 6A), suggesting that the deacetylation of MCCC2 at K269 facilitated its formation of the complex with MCCC1. We next tested if the MCCC2 activity is altered with its deacetylation at K269 mediated by SIRT4 by performing an *in vitro* MCCC2 enzymatic activity assay based on the production of [^14^C] 3-methylglutaconyl-CoA from [^14^C] bicarbonate and 3-methylcrotonyl-CoA 33. The enzymatic activities of MCCC2^K269R^ in both the Huh-7 and PLC/PRF/5 cells with endogenous MCCC2 knocked out were significantly higher than those of WT and MCCC2^K269Q^ (Figure 6B). Consistent with the enhanced enzymatic activity of MCCC2^K269R^, overexpression of SIRT4 in Huh-7 and PLC/PRF/5 cells also led to increased MCCC activity, compared with the respective controls (Figure 6C).

To address if the enhanced MCCC enzymatic activity resulting from SIRT4-mediated MCCC2 deacetylation affects the levels of acetyl-CoA, an intermediated metabolite of leucine metabolism, we subsequently measured the levels of acetyl-CoA using the PicoProbe AcCoA assay kit. Consistent with the metabolomic data, overexpression of SIRT4 in both Huh-7 and PLC/PRF/5 cells increased the levels of acetyl-CoA significantly compared with the respective controls (Figure 6D). Moreover, forced expression of the deacetylated mimetic mutant K269R in both Huh-7 and PLC/PRF/5 cells also led to elevated levels of acetyl-CoA, compared with the cells ectopically expressing WT, and the acetylated mimetic mutant K269Q (Figure 6E).

Collectively, these data demonstrate that the deacetylation of MCCC2 at K269 mediated by SIRT4 contributes to the formation or stability of MCCC1/MCCC2 complex with enhanced MCCC enzymatic activity, and thus increases the production of acetyl-CoA.

### Acetyl-CoA promotes H3K27 acetylation and stemness of HCC at low doses

Acetyl-CoA is a donor for the acetylation of a variety of proteins including histones, which are involved in the epigenetic regulation of gene expression and have been linked to the regulation of the stemness of embryonic stem cells 34, 35. Hence, we decided to address whether acetyl-CoA itself plays any role in the determination of stem cell-like properties of HCC TICs. Since cells are impermeable to acetyl-CoA, we used streptolysin-O (SLO) to deliver different concentrations of exogenous acetyl-CoA into the cells. SLO-assisted intracellular delivery of acetyl-CoA increased the expression of H3K27ac, ABCG2, BMI1, and NANOG, as well as the spheroid formation efficiencies of both the Huh-7 and PLC/PRF/5 cell lines at doses of 1, and 2 µM. However, the promoting roles of acetyl-CoA in HCC TIC properties diminished at a dose of 10 µM (Figure 6F-H), suggesting that the roles of acetyl-CoA in HCC TICs depend on its concentration within the cells.

### SIRT4 has opposite roles in α2δ1^-^ and α2δ1^+^ fractions of HCC cells

The finding that low and high doses of exogenous acetyl-CoA had distinguishable roles in the determination of H3K27ac level and stemness of HCC TICs promoted us to hypothesize that the role of SIRT4 in tumor, which determined the amount of acetyl-CoA, might be dependent on its levels in the cells. To test this hypothesis, we overexpressed SIRT4 in purified α2δ1^-^ and α2δ1^+^ PLC/PRF/5 cells with low and high SIRT4 expression, respectively, and inoculated 10^5^ cells per site subcutaneously into NOD/SCID mice to compare their tumorigenicity side by side. Overexpression of SIRT4 in the α2δ1^-^ PLC/PRF/5 cells enhanced the tumorigenicity of these cells, which was consistent with our above conclusion that SIRT4 was an oncogene (Figure 6I and J, Figure S2C), whereas forced expression of SIRT4 in α2δ1^+^ ones resulted in remarkable suppression of the tumorigenicity of the cells (Figure 6K and L, Figure S2 D), supporting its tumor suppressor role in α2δ1^+^ HCC cells. These data demonstrate that SIRT4 plays dual roles in tumorigenesis which depend on its levels within the cells.

### SIRT4-mediated deacetylation of MCCC2-K269 modulates H3K27 acetylation and gene expression

**Figure 7:**
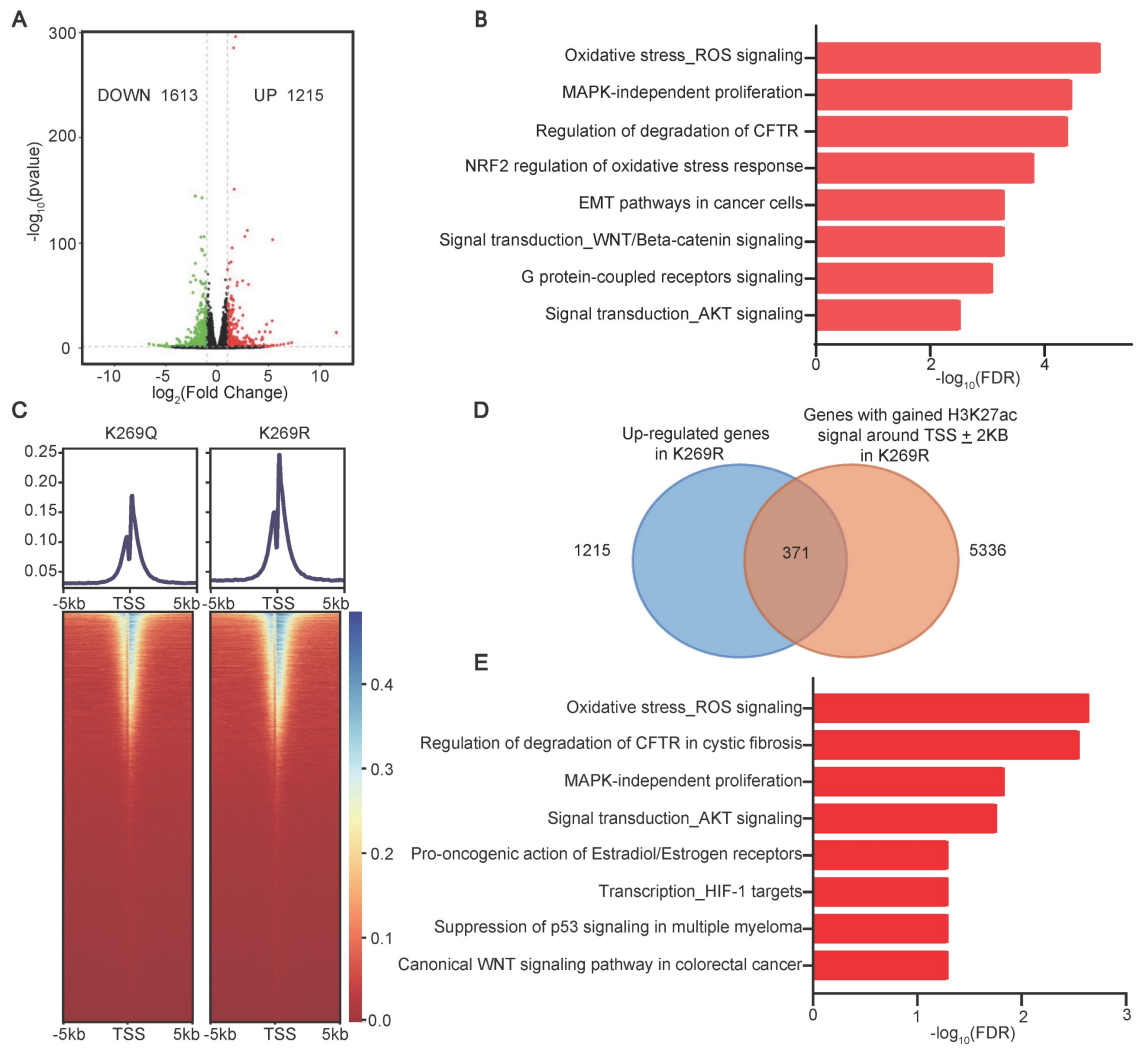
** Deacetylation of MCCC2-K269 resulted in epigenetic reprogramming. (A)** Volcano plot showing the differentially expressed genes (DEG) in Huh-7 cells overexpressing MCCC2^K269R^ compared with the MCCC2^K269Q^ ones (∣Fold Change∣˃1.5, pvalue˂0.05). **(B)** Top pathways (FDR<0.05) identified by Metacore Functional Ontology Enrichment among the up-regulated genes in Huh-7 cells overexpressing MCCC2^K269R^ compared with the MCCC2^K269Q^ ones. **(C)** Heatmap showing the occupancy of genome-wide H3K27ac peaks in a ± 5 kb window surrounding the transcription start sites (TSS) in MCCC2^K269R^ Huh-7 cells and MCCC2^K269Q^ ones. **(D)** Venn diagram showing the overlap of upregulated genes in RNA-seq and H3K27ac ChIP-seq in MCCC2^K269R^ Huh-7 cells versus MCCC2^K269Q^ ones. **(E)** Top pathways (FDR<0.05) identified by Metacore Functional Ontology Enrichment among overlapping genes described in **D**.

Our finding that SIRT4-mediated MCCC2-K269 deacetylation resulted in increased acetyl-CoA production which induced H3K27 acetylation and TIC properties prompted us to hypothesize that SIRT4-mediated MCCC2-K269 deacetylation prompted HCC TIC properties by modulating the pathway(s) involved in stemness regulation via H3K27ac. As expected, forced expression of SIRT4 and the deacetylated mimetic mutant K269R of MCCC2 were able to increase the levels of H3K27ac in both Huh-7 and PLC/PRF/5 cell lines, compared with the respective controls (Figure S2E). RNA sequencing in Huh-7 cells stably expressing MCCC2^K269R^ or MCCC2^K269Q^ revealed that a total of 1215 genes were up-regulated and 1613 genes were down-regulated in the cells expressing MCCC2^K269R^ compared with those expressing MCCC2^K269Q^ (Figure 7A, Table S5). MetaCore enrichment analysis of the differentially expressed genes showed that the up-regulated transcripts were most enriched in many pathways such as WNT/β-catenin signaling, epithelial-mesenchymal transition (EMT), and AKT signaling (Figure 7B). In particular, those genes involved in embryonic stem cell development including *MYC*, *WNT*, *JAK2*, *SOX4* and *TAF5L*, were also among the MCCC2^K269R^-upregulated genes. Furthermore, ChIP-seq experiments using H3K27ac identified 14,800 and 13,512 peaks, which were highly enriched near the transcription start sites (TSSs), in the MCCC2^269R^ and MCCC2^269Q^ cells, respectively (Figure 7C). Integration of transcriptome sequencing with ChIP-seq data identified a total of 371 genes with increased expression levels and elevated H3K27ac modification in MCCC2^K269R^-expressing cells (Figure 7D, Table S6). Those genes were enriched in many pathways such as AKT signaling and WNT signaling as revealed by MetaCore enrichment analysis (Figure 7E). In addition, those genes involved in embryonic stem cell development such as *MYC*,* JAK2* and *TAF5L* were encompassed in this set, underscoring the potential of SIRT4-mediated deacetylation of MCCC2 at K269 to augment the stem cell-like properties of HCC.

## Discussion

In this study, we identified a previously unappreciated role of SIRT4, which is a downstream target of α2δ1-mediated calcium signaling, in promoting the stem cell-like properties and invasiveness of HCC cells through deacetylating MCCC2 at K269 to form MCCC1/MCCC2 enzymatic complex with enhanced enzymatic activity. Furthermore, both forced expression of SIRT4 and the deacetylated mimetic mutant MCCC2^K269R^ increased the production of acetyl-CoA, resulting in elevated levels of H3K27ac which is generally recognized as a marker for active enhancers and promoters 36. Hence, our study links α2δ1-mediated calcium signaling to SIRT4-mediated epigenetic regulation of the stem cell-like properties via enhancing the production of the metabolic intermediate acetyl-CoA, uncovering a novel signaling pathway in determining the properties of HCC TICs.

Many previous studies have identified SIRT4 as a tumor suppressor 16, 37, although there were reports indicating that SIRT4 was upregulated in several cancer tissues such as breast and esophageal cancer 19, 20, and it was required for cell survival during oncogene-induced transformation 38. Our current study supports its oncogenic role in promoting stemness of HCC cells by enhancing the production of acetyl-CoA. However, it could serve as a tumor suppressor when it is overexpressed in the cancer cells expressing high levels of SIRT4 such as α2δ1^+^ HCC TICs, as demonstrated herein (Figure 6I-J, Figure S2C and D). The dual roles of SIRT4 in HCC hence are cell-context and its expression level dependent. Such opposite roles of SIRT4 in HCC were likely attributed to its function in controlling the production of acetyl-CoA, which exhibits tumor-promoting or suppressing roles dependent on its concentrations as showed in this study, albeit further studies are required to address the underlying mechanisms and accurately quantify the amount of acetyl-CoA in different contexts. Nevertheless, our study emphasizes the importance of both the gene and metabolite doses in their roles in cancer, and identifies SIRT4 as an oncogene that might be targeted by small molecule inhibitors, antisense oligonucleotides, etc. for the treatment of HCC.

Acetyl-CoA is an essential cofactor for histone acetyltransferases (HATs) which catalyze the transfer of an acetyl group from acetyl-CoA to the lysine ε-amino groups on the N-terminal tails of histones when it is transported into nucleus 39. The acetylation of histones at multiple sites including H3K27 could lead to chromosome conformational change, altering the accessibility of transcriptional machinery or serving as histone codes to specifically regulate gene expression. Specifically, as reported in the literature, H3K27ac dynamics modulated interaction frequency between regulatory regions and could lead to allele-specific chromatin configurations to sustain oncogene expression 40. Our study identified SIRT4 as a crucial factor for enhancing acetyl-CoA production, increasing H3K27ac level, activating the expression of the genes involved in embryonic stem cell development such as *MYC*,* JAK2*, and *TAF5L*, as well as the signaling transduction such as* AKT and WNT*, and promoting the acquisition of stem cell-like properties via directly deacetylating MCCC2, an enzyme involved in leucine catabolism. Furthermore, high concentrations of acetyl-CoA failed to increase the H3K27ac level and were thus unable to promote the stem cell-like traits, which might use a mechanism that inhibits the activity of the enzymes that modulate H3K27ac, either in an allosteric manner or by altering substrate availability, as proposed in the literature 41. Hence, the ability of acetyl-CoA to enhance the acetylation of H3K27, which epigenetically modulates the specificity of the expression of genes involved in the acquisition of TIC traits, represents a key mechanism underlying its role in promoting HCC TIC properties and progression.

Acetylation/deacetylation of lysine residue represents one of the key posttranslational modifications in the regulation of the enzymatic activities involved in metabolic reprogramming in many physiological and pathological processes, including cancer occurrence and progression 42-44. Previous work identified the link between SIRT4 and BCAA catabolism through modulation of the activity of methylcrotonyl-CoA carboxylase, an enzyme involved in the leucine oxidation pathway, in liver and adipogenesis 7, 33. Specifically, multiple acylated lysine sites, including glutarylated, methylglutarylated, 3-methylglutaconylated, hydroxymethylglutarylated, succinylated, and acetylated modifications in the subunit 1 of the MCCC complex, were suggested to be responsible for decreased MCCC enzymatic activities, while significantly fewer modifications were detected in the MCCC2 subunit in mouse livers with SIRT4 knockout 7. Our study demonstrates that it is the acetylated status of MCCC2 at K269 that determines the MCCC1/MCCC2 complex formation and MCCC enzymatic activity in HCC, although how this modification specifically affects its interaction with MCCC1 and complex stability remains to be addressed. It seems unlikely that SIRT4 affects the acetylation level of MCCC1 as demonstrated here in Figure 3G, which is contradictory to the previous report 7.

It is worth noting that the intracellular levels of valine and leucine were upregulated in SIRT4-overexpressing HCC cells as demonstrated by metabolomic analysis, which might be caused by higher uptake of these amino acids as demonstrated by Zaganjor et al 33. Our study unravels a critical role of SIRT4-mediated leucine metabolism in the acquisition and subsequent maintenance of HCC TIC properties, however, the mechanism(s) underlying enhanced uptake of these amino acids resulting from SIRT4 overexpression remains to be addressed.

In summary, our study reveals novel roles of SIRT4 in controlling acetyl-CoA production and prompting the stem cell-like properties of HCC. Furthermore, the expression of SIRT4 in HCC is predictive of short disease-free and overall survival of the patients. Future study is warranted to address if targeting SIRT4 using its small molecule inhibitor could have therapeutic value in HCC. Nevertheless, our findings may improve our understanding of the mechanisms underlying the acquisition and maintenance of HCC TIC properties, and contribute to the discovery of prognostic markers and therapeutic targets of HCC.

## Supplementary Material

Supplementary figures and tables.

## Figures and Tables

**Table 1:** The tumorigenicity of the indicated cells with SIRT4 or SIRT4^H161Y^ overexpression.

Group	Group	1000	1000	100	100	Frequency of tumorigenic cells (95% CI)	*P* value	
Huh-7 α2δ1^-^	Vector	Vector	2/5	3/5	1/830(1/2308-1/299)	1/830(1/2308-1/299)		
Huh-7 α2δ1^-^	SIRT4	SIRT4	5/5	5/5	1/1(1/125-1)	1/1(1/125-1)	1.2E-5^a^	1.2E-5^a^
Huh-7 α2δ1^-^	SIRT4^H161Y^	SIRT4^H161Y^	3/5	2/5	1/711(1/1928-1/262)	1/711(1/1928-1/262)	0.814^a^	0.814^a^
PLC/PRF/5 α2δ1^-^	Vector	Vector	2/5	3/5	1/830(1/2308-1/299)	1/830(1/2308-1/299)		
PLC/PRF/5 α2δ1^-^	SIRT4	SIRT4	5/5	5/5	1/1(1/125-1)	1/1(1/125-1)	1.2E-5^a^	1.2E-5^a^
PLC/PRF/5 α2δ1^-^	SIRT4^H161Y^	SIRT4^H161Y^	2/5	2/5	1/1061(1/3128-1/360)	1/1061(1/3128-1/360)	0.719^a^	0.719^a^

^a^Compared with respective control group.

**Table 2:** The tumorigenicity of the MCCC2-KO cells that ectopically expressed the indicated sgRNA-resistant MCCC2 mutants

Group	Group	1000	1000	100	Frequency of tumorigenic cells (95% CI)	*P* value
Huh-7	MCCC2-WT	MCCC2-WT	3/5	2/5	1/711(1/1928-1/262)	
Huh-7	MCCC2-269R	MCCC2-269R	5/5	5/5	1/1(1/125-1)	3.3E-5^a^
Huh-7	MCCC2-269Q	MCCC2-269Q	3/5	1/5	1/921(1/2617-1/324)	0.711^a^
Huh-7	MCCC2-141R	MCCC2-141R	3/5	2/5	1/711(1/1928-1/262)	1^a^
Huh-7	MCCC2-141Q	MCCC2-141Q	3/5	2/5	1/711(1/1928-1/262)	1^a^
PLC/PRF/5	MCCC2-WT	MCCC2-WT	2/5	2/5	1/1061(1/3128-1/360)	
PLC/PRF/5	MCCC2-269R	MCCC2-269R	5/5	5/5	1/1(1/125-1)	4.7E-6^a^
PLC/PRF/5	MCCC2-269Q	MCCC2-269Q	3/5	2/5	1/711(1/1928-1/262)	0.562^a^
PLC/PRF/5	MCCC2-141R	MCCC2-141R	3/5	3/5	1/571(1/1519-1/215)	0.349^a^
PLC/PRF/5	MCCC2-141Q	MCCC2-141Q	2/5	2/5	1/1061(1/3128-1/360)	1^a^

^a^Compared with the respective MCCC2-Wild type group.

## Abbreviations

HCC: hepatocellular carcinoma
TIC: tumor-initiating cell
MCCC2: methylcrotonyl-CoA carboxylase subunit 2
MCCC1: methylcrotonyl-CoA carboxylase subunit 1
MCCC: 3-methylcrotonyl-CoA carboxylase
BCAA: branched-chain amino acid
VGCC: voltage-gated calcium channels
SDS-PAGE: sodium dodecyl sulfate-polyacrylamide gel electrophoresis
FACS: fluorescence-activated cell sorting
Co-IP: co-immunoprecipitation
shRNA: short hairpin RNA
sgRNA: single guide RNA
NOD/SCID: nonobese diabetic/severe combined immunodeficient
OE: overexpression
DFS: Disease-Free Survival
OS: Overall Survival
TCGA: The Cancer Genomic Atlas
YAP: Yes-associated protein
SLO: streptolysin-O
HATs: histone acetyltransferases
