# Supplementary material for: SIRT4 Controls Acetyl-CoA Synthesis to Promote Stemness and Invasiveness of Hepatocellular Carcinoma through Deacetylating MCCC2
Source: Int J Biol Sci. 2025 Apr 21;21(7):2973–90. doi: 10.7150/ijbs.99004 (PMC12080386; doi:10.7150/ijbs.99004)
Supplement: Supplementary file 1 — Supplementary figures and tables. [file ijbsv21p2973s1.zip › Supplementary Materials/Supplementary Materials.pdf]

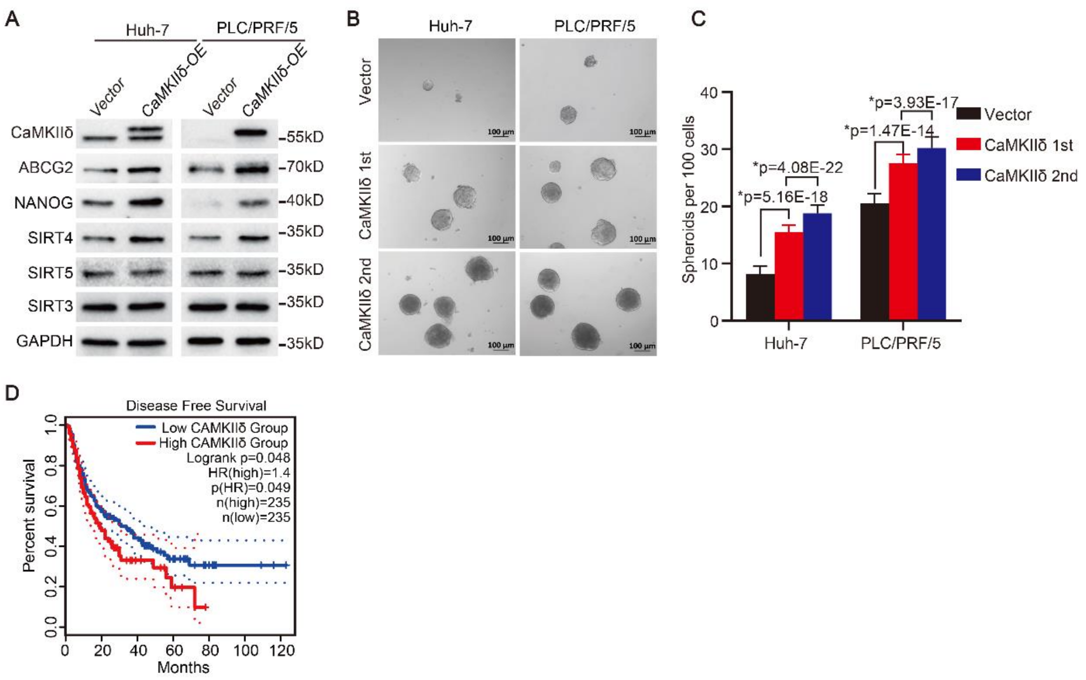

Figure S1 CaMKIIδ promotes HCC TICs and is associated with poor prognosis.

(A) Western blotting results showing the expression of the indicated molecules in indicated cells overexpressing *CaMKIIδ*. (**B and C**) Phase contrast micrographs **(B)** and histograms **(C)** demonstrating primary and serially passaged spheres formed by the indicated cells after forced expression of *CaMKIIδ*. Data represent mean ± SD of 3 independent experiments. *Two-tailed Student's *t* test. Scale bars = 100 μm. **(D)** Kaplan-Meier survival analysis for the expression of *CaMKIIδ* mRNA in HCC patients was performed using an online tool (http://gepia2.cancer-pku.cn/).

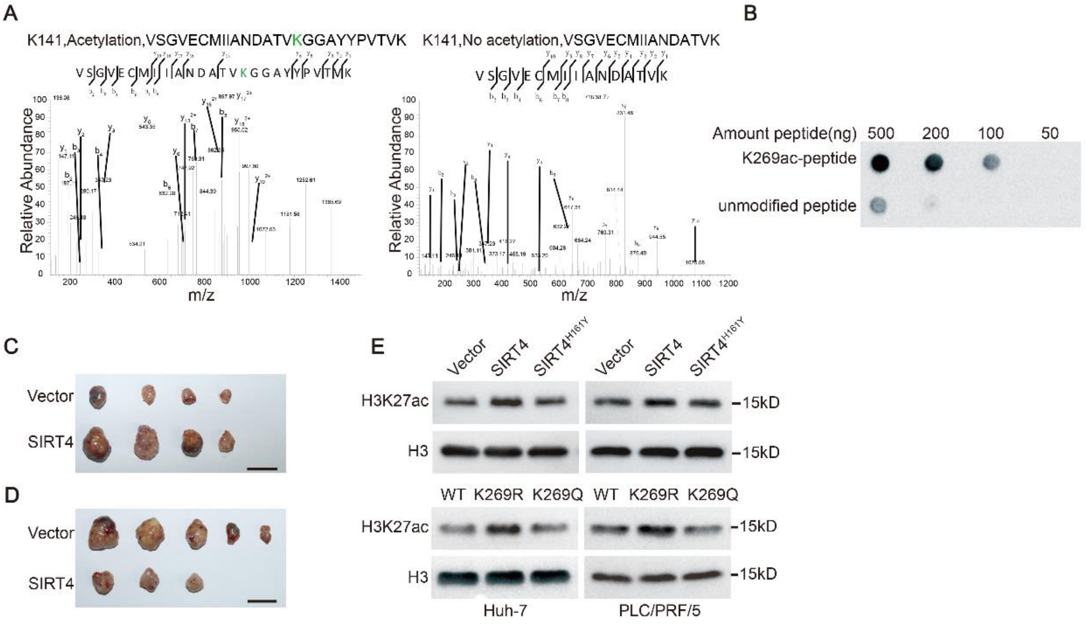

Figure S2

(A) Mass spectrometry analysis of the acetylation sites of MCCC2-Flag immunoprecipitated in Figure 3I. **(B)** The specificity of the antibody against acetyl-K269 of MCCC2 was determined by dot blot assay. **(C and D)** Photograph of dissected tumors of the tumors formed by $\alpha2\delta1^-$ **(C)** (n=4) or $\alpha2\delta1^+$ **(D)** (n=5) PLC/PRF/5 cells overexpressing SIRT4 or empty vector in NOD/SCID mice. **(E)** Western blotting analysis of the acetylation levels of H3K27 in indicated cells.

Table S1. Information for Antibodies

Name.	Vender	Cat No.	Species	Dilution
ABCG2	Abcam	ab108312	Rabbit monoclonal IgG	1:3000
BMI1	Abcam	ab126783	Rabbit monoclonal IgG	1:5000
NANOG	Cell Signaling	4903S	Rabbit monoclonal IgG	1:3000
α2δ1	Novus	NB120-2864	Mouse monoclonal IgG	1:3000
α2δ1	Sigma	HPA008213	Rabbit monoclonal IgG	1:3000
SIRT4	Sigma	HPA029691	Rabbit polyclonal IgG	1:3000
SIRT3	Proteintech	10099-1-AP	Rabbit polyclonal IgG	1:3000
SIRT5	Abcam	ab259967	Rabbit monoclonal IgG	1:3000
MCCC2	Proteintech	12117-1-AP	Rabbit polyclonal IgG	1:3000
MCCC1	Proteintech	14861-1-AP	Rabbit polyclonal IgG	1:3000
CaMKIIδ	Abcam	ab181052	Rabbit monoclonal IgG	1:3000
H3	Beyotime	AH433	Rabbit polyclonal IgG	1:3000
H3K27ac	Abcam	ab4729	Rabbit polyclonal IgG	1:3000
Flag	Cell Signaling	14793S	Rabbit monoclonal IgG	1:5000
HA	Cell Signaling	3724S	Rabbit monoclonal IgG	1:3000
Acetylated-Lysine	Cell Signaling	9441S	Rabbit monoclonal IgG	1:3000
GAPDH	Cell Signaling	2118S	Rabbit monoclonal IgG	1:10000

Table S2. Sequences for PCR Primers and shRNA/sgRNA constructs

Name	Primer	Sequences
SIRT4 Wild-type	Forward	5'-CGGGATCCATGAAGATGAGCTTTGCGTTGAC-3'
	Reverse	5'-CCGCTCGAGGCATGGGTCTATCAAAGGCAGCAAC-3'
SIRT4-H161Y	Forward	5'-CGCCTGACAGAGCTCTACGGATGCATGG-3'
	Reverse	5'-CTGTCCATGCATCCGTAGAGCTCTGTCAGG-3'
SIRT4 shRNA-1	Forward	5'-gatccCCCGATTGCAATACTGAACATcttcctgtcagaATGTTCAGTATTGCAATCGGGtttttg-3'
	Reverse	5'-aattcaaaaaCCCGATTGCAATACTGAACATtctgacaggaagATGTTCAGTATTGCAATCGGGg-3'
SIRT4 shRNA-2	Forward	5'-gatccCCGTGCTCGAAAGCCTCCATTcttcctgtcagaAATGGAGGCTTTCGAGCACGGtttttg-3'
	Reverse	5'-aattcaaaaaCCGTGCTCGAAAGCCTCCATTtctgacaggaagAATGGAGGCTTTCGAGCACGGg-3'
Scramble shRNA	Forward	5'-gatccGCGAGAAGCGCGATCACATGTTCAAGAGACATGTGATCGCGCTTCTCGtttttg-3'
	Reverse	5'-aattcaaaaaACGAGAAGCGATCACATGTCTCTTGAACATGTGATCGCGCTTCTCGCg-3'
SIRT3 Wild-type	Forward	5'-CGCGGATCCATGGTGGGGGCCGGCATCAG-3'
	Reverse	5'-CCGCTCGAGTTTGTCTGGTCCATCAAGCTTCCCA-3'
MCCC2 Wild-type	Forward	5'-CGGGATCCATGTGGGCCGTCCTGAGGTTA-3'
	Reverse	5'-CCGCTCGAGCATCCTGAAGATACCGAAGT-3'
MCCC1 Wild-type	Forward	5'-CGGGATCCATGAAGTACACAACAGCCACA-3'
	Reverse	5'-ATCTTCCGATTCCCTTTTGTCTGATTCTTCCT-3'
MCCC2 sgRNA	Forward	5'-CACCGTTGACAATCTCATAGACCCA-3'
	Reverse	5'-AAACTGGGTCTATGAGATTGTCAAC-3'
MCCC2-141R	Forward	5'-CAATGATGCCACCGTCAGAGGAGGTGCCT-3'
	Reverse	5'-AGGCACCTCCTCTGACGGTGGCATCATTG-3'
MCCC2-141Q	Forward	5'-CAATGATGCCACCGTCCAAGGAGGTGCC-3'
	Reverse	5'-GGCACCTCCTTGGACGGTGGCATCATTG-3'
MCCC2-269R	Forward	5'-GGAGGTGCTGATCTTCATTGCAGAAGGTCT-3'
	Reverse	5'-CAGACCTTCTGCAATGAAGATCAGCACCT-3'
MCCC2-269Q	Forward	5'-GGTGCTGATCTTCATTGCAGACAGTCTGGA-3'
	Reverse	5'-TCCAGACTGTCTGCAATGAAGATCAGCACC-3'

Table S3. Correlation between the clinicopathologic characteristics and expression of SIRT4 protein in hepatocellular carcinoma

Variable	SIRT4 expression		Chi square value	*P* value[a]
	Low	High		
Sex				
Male	28	28	0.8634	0.3528
Female	6	3		
Age				
≤60	24	26	1.612	0.2043
>60	10	5		
Hepatic cirrhosis				
Absent	14	8	1.711	0.1909
Present	20	23		
Size				
≤5 cm	21	10	5.659	0.0174*
>5 cm	13	21		
Venous invasion				
Absent	26	19	1.754	0.1854
Present	8	12		
Invasion				
Absent	31	29	0.1285	0.7200
Present	3	2		
Recurrence				
Early [b]	5	14	8.374	0.0038**
Late [c]	29	15		
AFP				
>400 μg/L	6	14	6.330	0.0119*
≤400 μg/L	24	13		

Note: a, Chi-Square Tests.

b, Recurrences occur within 2 years of diagnosis.

c, No recurrences within 2 years of diagnosis.
